# Correction: Radman et al. Prevalence of Key Modifiable Cardiovascular Risk Factors among Urban Adolescents: The CRO-PALS Study. *Int. J. Environ. Res. Public Health* 2020, *17*, 3162

**DOI:** 10.3390/ijerph20217003

**Published:** 2023-10-31

**Authors:** Ivan Radman, Maroje Sorić, Marjeta Mišigoj-Duraković

**Affiliations:** 1 Faculty of Kinesiology, University of Zagreb, 10000 Zagreb, Croatia; maroje.soric@kif.unizg.hr (M.S.); marjeta.misigoj-durakovic@kif.unizg.hr (M.M.-D.); 2 Faculty of Sport, University of Ljubljana, 1000 Ljubljana, Slovenia

## 1. Error in Figure

The authors regret to acknowledge that a mistake has been made in the original article [1], as published. Specifically, an error was made in the Results section and Figure 1, in the “high screen time” histogram graph. Unfortunately, it was observed that the true screen time values were erroneously divided by 7, and thereby caused the displayed high screen time prevalence in the histogram graph to decrease from the true value to a large extent. Therefore, both Figure 1 and the interpretation of the results need to be revised. The corrected Figure 1 appears bellow, and the corrected sentences are stated in the Text Correction.

**Figure 1:**
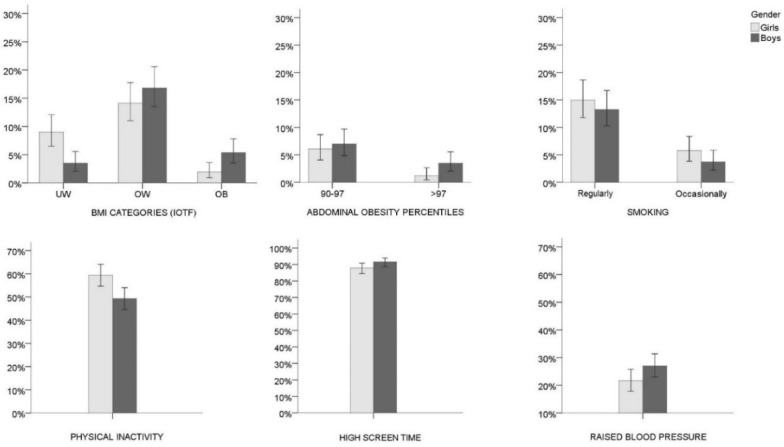
Prevalence of the body mass index (BMI) categories (girls N = 410; boys N = 428), abdominal obesity percentile categories (girls N = 411; boys N = 429), smoking (girls N = 414; boys N = 429), physical inactivity (girls N = 414; boys N = 430), high screen time (girls N = 414; boys N = 429), and raised blood pressure (girls N = 416; boys N = 429) stratified by sex. Error bars represent 95% confidence levels; IOTF = International Obesity Task Force; UW = underweight; OW = overweight; OB = obese. Note: Girls and boys were allocated to the BMI categories according to the IOTF cut-off values proposed by Cole and Lobstein, 2012 [27], and to the abdominal obesity categories based on cut-off values of 90th and 97th centiles for German adolescents of respective age between 2003–2007 (Kromeyer-Hauschild et al., 2010 [28]); physical inactivity refers to <60 min of moderate-to-vigorous physical activity per day; high screen time refers to >120 min of screen related activities per day; Raised blood pressure refers to systolic and/or diastolic blood pressure above the 95th percentile for age, sex, and height as described by Falkner et al., 2001 [31].

## 2. Text Correction

In a light of changes in results and Figure 1, the interpretation of the results has been revised. All the corresponding sentences have been revised, as listed below.

First, authors revised the Abstract section revealing the results: “The outcomes suggest that more than one half did not meet the recommended daily physical activity (girls 59.4%; boys 45.5%), a vast majority exceeded 2 h of screen time per day (girls 87.9%; boys 91.6%), and one quarter had high blood pressure (girls 21.6%; boys 27.0%)”.

Second, authors corrected the Results section, second paragraph: “A vast majority of both boys and girls spent more than 120 min/day on average in front of screens (91.6 (88.7–94.0)% and 87.9 (84.5–90.8)%)”.

Third, we made three corrections in the Discussion section. In the first paragraph we changed the sentence: “The main findings suggest that more than one half did not meet recommended minimum of daily physical activity, nine tenths spent more than two hours in front of the screen per day, and about one quarter of observed adolescents had high blood pressure”. Two additional changes were made in the second paragraph, the sentence: “Current results indicate a lower rate of insufficient physical activity (59.7% girls and 45.5% boys) and considerably higher rate of excess screen time (both sexes nearly 90%) in the studied population in comparison to worldwide reports” and the further sentence: “Conversely, the incidence of nearly 90% excessive screen time in the current study population was considerably higher than the 41% reported in 2015 for a similar population in the neighboring Slovenian capital Ljubljana [48]”.

The authors state that the scientific conclusions are unaffected. This correction was approved by the Academic Editor. The original publication has also been updated.
